# Non-pharmacological interventions for vascular health and the role of the endothelium

**DOI:** 10.1007/s00421-022-05041-y

**Published:** 2022-09-23

**Authors:** Samuel R. C. Weaver, Catarina Rendeiro, Rebekah A. I. Lucas, N. Timothy Cable, Tom E. Nightingale, Helen M. McGettrick, Samuel J. E. Lucas

**Affiliations:** 1 School of Sport, Exercise and Rehabilitation Sciences, College of Life and Environmental Sciences, University of Birmingham, Birmingham, UK; 2 Centre for Human Brain Health, University of Birmingham, Birmingham, UK; 3 Institute of Sport, Manchester Metropolitan University, Manchester, UK; 4 Institute of Inflammation and Ageing, College of Medical and Dental Sciences, University of Birmingham, Birmingham, UK

**Keywords:** Vascular function, Non-pharmacological intervention, Exercise, Endothelial, Heat, Hypoxia, Ischemic preconditioning

## Abstract

The most common non-pharmacological intervention for both peripheral and cerebral vascular health is regular physical activity (e.g., exercise training), which improves function across a range of exercise intensities and modalities. Numerous non-exercising approaches have also been suggested to improved vascular function, including repeated ischemic preconditioning (IPC); heat therapy such as hot water bathing and sauna; and pneumatic compression. Chronic adaptive responses have been observed across a number of these approaches, yet the precise mechanisms that underlie these effects in humans are not fully understood. Acute increases in blood flow and circulating signalling factors that induce responses in endothelial function are likely to be key moderators driving these adaptations. While the impact on circulating factors and environmental mechanisms for adaptation may vary between approaches, in essence, they all centre around acutely elevating blood flow throughout the circulation and stimulating improved endothelium-dependent vascular function and ultimately vascular health. Here, we review our current understanding of the mechanisms driving endothelial adaptation to repeated exposure to elevated blood flow, and the interplay between this response and changes in circulating factors. In addition, we will consider the limitations in our current knowledge base and how these may be best addressed through the selection of more physiologically relevant experimental models and research. Ultimately, improving our understanding of the unique impact that non-pharmacological interventions have on the vasculature will allow us to develop superior strategies to tackle declining vascular function across the lifespan, prevent avoidable vascular-related disease, and alleviate dependency on drug-based interventions.

## General introduction and scope of review

As the highway connecting every organ within the human body, the vascular system plays a unique and central role in maintaining health, while simultaneously holding the potential to disrupt multiple regions when not optimally functioning. As a result, vascular dysfunction plays a central role in disease progressions throughout the body, including in cardiovascular disease, stroke, and dementia (Toth et al. 2017; Kraus et al. 2019). The dangers of poor vascular health are becoming increasingly apparent in the modern world, as lifestyle-related factors play an ever more central role in all-cause mortality, and both communicable and cancer-related deaths are replaced by cardiovascular disease as the number one cause of mortality across the lifespan (Roth et al. 2020). The deleterious impact that poor vascular health has on an individual’s systemic health and quality of life, in addition to the escalating global burden of disease caused by poor vascular health, makes it imperative that effective and accessible interventions are prioritised to minimise the impact of vascular decline.

This review aims to examine the potential for non-pharmacological interventions to improve vascular function through acute changes in blood flow and its impact on the endothelium. The mechanisms driving these beneficial responses will be defined, alongside the potential for changes in the circulating molecular environment to modulate flow-dependent responses. In addition, we will look to explore the limitations in our current knowledge and understanding of how changes in flow affect the endothelium, particularly concerning the impact of non-pharmacological interventions. This will allow the development of more intervention-specific in vitro research methods and, as a result, reduce our reliance on the translation of results from disease and dysfunctional flow models. Dietary interventions will not be discussed within the scope of this review, as while these interventions have the potential to improve vascular function they act primarily through alternative mechanisms to those seen in exercise and other flow-dependent strategies and have been extensively reviewed elsewhere (Rees et al. 2018; Brandhorst and Longo 2019; Weaver et al. 2020). Additionally, due to a lack of in vivo studies in humans, it is not possible to explore responses within smooth muscle cells and further work is required in this area.

## Vascular responses to non-pharmacological interventions

The cumulative nature of vascular decline, coupled with the cost and potential health implications involved in drug-based treatment, has led to growing interest in non-pharmacological interventions strategies. These strategies have the added benefit of being applicable in a community setting, without the need for clinical diagnosis and can be utilised in a preventative manner, as has been shown in the effective reduction of other lifestyle-related diseases such as type II diabetes (Schwarz et al. 2012). Moreover, when utilised as “lifestyle” interventions, non-pharmacological approaches can have a holistic effect in improving both vascular function and general health (Dagogo-Jack et al. 2010). These approaches include, but are not limited to, exercise-based interventions, heat therapy (e.g., hot water or sauna bathing), hypoxia-based interventions, ischemic preconditioning, and pneumatic compression (Allen and Morey 2010).

Within the context of the vasculature, the responses to non-pharmacological interventions can be broadly separated into the direct response to changes in blood flow (i.e., flow-dependent) within a given vessel and the impact that flow-independent changes in the circulating environment can have on the endothelium. This review will explore the isolated and combined effects of these two adaptive pathways, both within and between intervention approaches.

### Exercise

As physical activity plays such a central role in vascular health, it is of little surprise that exercise-based interventions are one of the most commonly utilised non-pharmacological approaches for improving vascular function (Green and Smith 2018). While the impact of exercise is widespread and can result in changes to a large number of circulating factors with vascular effects (Fiuza-Luces et al. 2018), it is the impact it has on blood flow and the resulting shear stress (frictional force exerted on the vessel wall) and cyclic strain (repetitive/beat-to-beat stretching force) within the vasculature that appear central to the benefits seen in vascular health (Green et al. 2017). Indeed, increases in flow provide an essential stress stimulus for vascular responses to exercise, as highlighted when restricting exercise-induced flow elevation limits subsequent adaptive improvements in flow-mediated dilation (FMD) (Tinken et al. 2010; Birk et al. 2012). Additionally, the changes in FMD following acute exercise have also been shown to correlate with basal changes seen following repeated bouts of exercise (i.e., training), demonstrating the role that transient responses to flow play in adaptive responses to interventions (Dawson et al. 2018).

The central role of flow in driving beneficial adaptations has been well demonstrated in the context of exercise, with evidence demonstrating an elevation in blood flow throughout the body during exercise (Padilla et al. 2011). While the specific flow response is highly dependent on the choice of protocol (e.g., exercise modality and intensity; Furlong et al. 2020), during the commonly studied protocol of moderate-intensity continuous cycling a twofold increase in brachial artery blood flow (Thijssen et al. 2009) and a 25% increase in middle cerebral artery blood velocity are seen (Weaver et al. 2021). Changes in circulating factors have also been identified as potential drivers of vascular change in response to exercise, although this area is less well studied than that of flow-related responses. Nevertheless, reported changes in circulating factors include exercise-induced elevations in angiogenic and vasodilatory factors, such as and vascular endothelial growth factor (VEGF) and transient elevations in inflammatory cytokine profile (Landers-Ramos et al. 2014). Interestingly, exercise has also been shown to induce significant, acute elevations in a number of factors linked to environment-based interventions, including heat shock proteins 72 (HSP72; Walsh et al. 2001), which could be linked to the interplay between the adaptive vascular benefits seen between intervention modalities (see “Flow-independent mechanisms”).

Exercise training (i.e., regular, repeated exercise bouts) facilitates longer term adaptations in vascular function, including improvements in basal brachial artery function as measured by FMD (Early et al. 2017) and vascular remodelling as evidenced in increased diameter and reduced media-intima thickness in the femoral artery (Dinenno et al. 2001). Similarly, in the cerebrovasculature, a recent meta-analysis highlighted that improved cerebrovascular function, as indexed by measures of cerebrovascular reactivity, is seen in individuals with higher fitness (Smith et al. 2021).

### Ischemic preconditioning

As well as exercise, elevations in blood flow are seen across a wide range of other non-pharmacological interventions, either through the specific design of interventions to alter blood flow, or through the manipulation of one or more factors that result in changes in flow. Of the intervention strategies that directly alter blood flow, the two supported by the greatest wealth of evidence are intermittent pneumatic compression and ischemic preconditioning. Ischemic preconditioning (IPC); characterised by short periods (4–5 min) of peripheral ischemia through inflation of a cuff to supra-systolic pressure, has also been linked to benefits in vascular function (Jones et al. 2014). Initially designed as a protective treatment against ischemic reperfusion injuries, the impact of brief periods of blood flow restriction and subsequent reperfusion have subsequently been shown to have a broader effect on endothelial function.

**Fig. 1:**
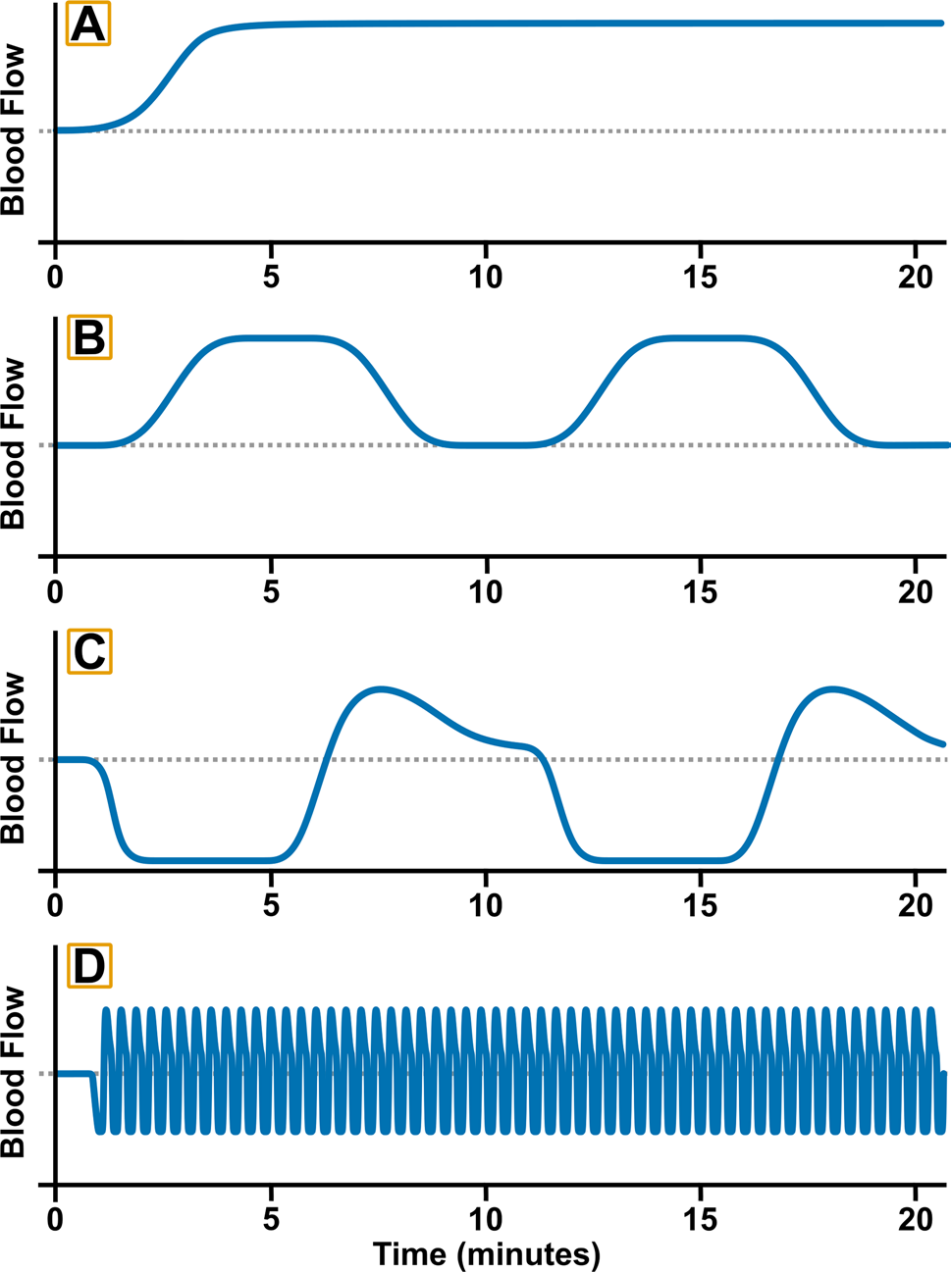
Representative flow patterns. Illustration of typical flow patterns seen across different intervention strategies, resting baseline is shown as a dashed line in all plots. **a** Prolonged elevated flow patterns as are commonly seen during interventions such as steady-state exercise or continuous exposure to heat stimuli (e.g., hot water bathing). **b** Interval or intermittent increases in flow, separated by a return to baseline, as is typical in interval-based exercise responses or in intermittent exposure to environmental stimuli. **c** Flow response seen during ischemic preconditioning, typified by a prolonged period of very low flow during the ischemic period, followed by a rapid elevation and steady return towards baseline during the cuff-release period. **d** Pneumatic compression induced flow patterns modelled on a low-frequency treatment pattern of repeated 4-s inflations and 16-s deflations

Acutely, alongside the direct manipulation of blood flow within the preconditioned limb (depicted in Fig. 1), IPC leads to vasodilation and increased blood flow in the contralateral limb (Enko et al. 2011). Over repeated treatments, IPC has been shown to increase endothelium-dependent forearm blood flow by ~ 30% in the preconditioned limb (Kimura et al. 2007), alongside significant improvements in both preconditioned and contralateral arm FMD (Jones et al. 2014). While systemic changes in flow and hyperemia have been shown to be central in driving contralateral limb adaptation (see “Flow-independent adaptation in vivo”), significant changes are also seen in the circulating environment that have the potential to provide additive benefits to vascular function and that could affect changes outside of peripheral arterial function (Thijssen et al. 2016). Crucially, while animal studies have identified increases in a range of vasoactive factors including systemic elevations in stromal-derived factor-1α, nitrite, microRNA-144 (Pickard et al. 2015), VEGF (Ueno et al. 2016), and interleukin 10 (IL-10; Cai et al. 2012), there is a scarcity of research exploring human in vivo circulating responses.

The majority of current research has focused on the impact of IPC protocols on arterial function in the reconditioned and contralateral limbs, while recent findings have shown conflicting results as to whether changes in dynamic cerebral autoregulation are seen following a single IPC stimulus (Guo et al. 2019; Carter et al. 2020). Future research should look to clarify these conflicting findings, as well as expanding our understanding of how the IPC stimulus may affect a broad range of vascular beds, especially the cerebrovascular.

### Pneumatic compression

Similarly, pneumatic compression-based interventions induce localised increases in blood flow using automated cuff-inflation systems, in which blood flow is increased through a cyclical pattern of inflation and deflation. This approach has been shown to drive positive responses in increasing shear rate in the compressed limb, with twofold elevations in mean shear rate during early compression and maintenance of elevated shear when compressions are carried out at a low frequency (3-s inflation, 17-s deflation) (Sheldon et al. 2012). While the nature of pneumatic compression innately alters blood flow, how this compression could drive changes in circulating microenvironment are relatively unexplored. However, studies within animal models have shown that compression protocols have the capacity to drive elevations in VEGF (Rivilis et al. 2002), suggesting that changes in the circulating microenvironment could also play a role in the vascular benefits of pneumatic compression.

Repeated exposure to pneumatic compression has shown significant improvements in markers of vascular function (Sheldon et al. 2013), as well as the potential to drive elevations in basal flow rate (Kakkos et al. 2005). However, to date, the majority of studies have been conducted across a broad range of clinical and aged populations, with a focus on vascular function specific to the population’s clinical outcomes of interest (Sheldon et al. 2013). This, combined with a lack of standardised compression protocols, means that further research is needed to fully understand the chronic benefits of pneumatic compression on vascular function, as well as allow comparison with other non-pharmacological interventions.

### Heat therapy

Acute elevations in flow can also be induced by the manipulation of the external environment, either through changes in temperature, humidity, and air composition (hypoxia); via immersion in water; or by a combination of these manipulations. The most common of these approaches are heat-based interventions that aim to induce a significant elevation in core temperature, which have subsequently shown the potential to drive adaptive improvements in endothelial function (Cheng and MacDonald 2018). As a result of increases in cardiac output and redistribution of blood in response to increases in core temperature, heat-based interventions have been shown to drive significant changes in blood flow, comparable to those seen in exercise, across a range of stimulus types, including hot water bathing (Thomas et al. 2017), sauna bathing (Li et al. 2020), and the utilisation of water perfused body suits (Chiesa et al. 2016). Similarly, despite a lack of skeletal muscle activation, passive heating interventions have also been shown to drive elevations in a number of vasoactive factors seen to increase during exercise, including IL-6 and HSP72 (Hoekstra et al. 2018).

Repeated exposure to heat-based interventions has also been shown to drive adaptive responses including elevation in basal cerebral blood flow/velocity alongside improvements in cerebrovascular conductance and FMD in the brachial artery (Bailey et al. 2016). While the majority of research into water immersion for vascular health has utilised hot water conditions, immersion in thermoneutral water also has the potential to increase acute peripheral blood flow (Ayme et al. 2014) and cerebral blood flow (Carter et al. 2014), thus providing a stimulus for flow-mediated adaptations regardless of water temperature (i.e., via hydrostatic pressure effects).

### Hypoxia-based interventions

Studies have also investigated the potential benefits of short-term hypoxia, in part due to its potential benefits in preconditioning against ischemic injury, and also for the potential benefits of low dose hypoxia in the wider vasculature (Navarrete-Opazo and Mitchell 2014). While the impact of exposure to hypoxia is systemic and thus affects a wide range of physiological systems, evidence does show that this stimulus can produce elevations in blood flow, through endothelium-dependent release of nitric oxide and prostaglandins (Dinenno 2016). Within the cerebrovasculature, acute hypoxia also induces changes in vascular function to maintain tissue oxygenation, including increases in blood flow in the vertebral artery (Ogoh et al. 2013) and vasodilation of the cerebral arteries (Wilson et al. 2011). Acute increases are also seen in a number of circulating factors including IL-6 (Hartmann et al. 2000), HSP72 (Taylor et al. 2010), and VEGF (Burki and Tetenta 2014).

Although these acute hypoxic responses show the potential for elevations in flow, to date, little is known about the efficacy of hypoxia as an intervention strategy. However, research in rodent models has shown positive responses to intermittent hypoxic training in both cognitive and cerebrovascular components of Alzheimer’s disease (Manukhina et al. 2016). Further research is needed to determine whether these responses will translate into hypoxic stimuli as an effective intervention for improving vascular function in humans.

### Summary

As research continues to advance our understanding of commonly used intervention approaches, the specific modality and methods used develop also. This includes the isolation of single components of an intervention, such as the use of thermoneutral water immersion mentioned above, or the combination of different approaches to maximise the potential stimulus for change; as demonstrated with aquatic treadmill-based exercise protocols (Parfitt et al. 2017) and the combining of exercise and hypoxia/blood flow restriction (Willis et al. 2019). Additionally, intervention strategies are beginning to be more specifically developed for clinical populations with reduced mobility, including the use of electrical of vibration-based stimulation in spinal cord patients (Games et al. 2015; Menendez et al. 2016). As we begin to better understand the acute impact of these interventions in vivo, it is important that we look to develop a clearer understanding of how these interventions may drive adaptive responses across the vasculature.

## Evidence for vascular adaptations in vivo

### Flow-dependent adaptation in vivo

Repeated exposure to many of these acute stimuli appears to stimulate similar adaptive responses, although the depth and breadth of our understanding of the processes driving these changes does vary significantly between interventions. Much like the weight of evidence for functional responses, the largest body of evidence surrounding the process and mechanisms of adaption can be found for exercise-based approaches. In a review of these processes, Green et al. (2017) explored the biphasic nature of adaptations to exercise. They highlighted that shorter intervention periods display a largely functional response phenotype, typified by increases in endothelium-dependent vasodilatory response and improvements in circulating inflammatory cytokines, oxidative stress markers, and vasodilatory factors. This initial response appears to then plateau at ~ 4 weeks, with further improvements being produced by way of structural changes in lumen diameter, wall:lumen ratio, and vessel elasticity (Green et al. 2017).

Across non-exercise strategies, the balance of functional and structural responses to interventions is best understood within the field of passive heating, as studies have demonstrated improvements in both endothelial functional responses and flow-mediated dilation, as well as structural improvements in elasticity and wall:lumen ratio over relatively short (8 week) intervention periods (Brunt et al. 2016b). Nonetheless, the amount of data on these responses is somewhat limited compared to the exercise context and there is still debate as to whether this pattern of response is likely to be seen across all heating modalities (Cheng and MacDonald 2018).

While further research is needed across the majority of flow inducing non-pharmacological interventions, our ability to quantify these responses in vivo is relatively well developed, particularly in relation the peripheral arteries of the legs and arms. This is due to well-established guidelines for utilisation of Doppler ultrasound to assess FMD of peripheral conduit arteries (Thijssen et al. 2011), alongside pulse wave velocity measures to assess arterial stiffness (Milan et al. 2019). Similarly, functional responses to interventions within the brain can be assessed through the effective utilisation of transcranial Doppler ultrasound (Willie et al. 2011), alongside more advanced techniques such as magnetic resonance imagery. In combination, these measures can begin to build a full picture of the acute and chronic responses to non-pharmacological interventions across a wide range of spatial and temporal resolutions within the brain (Tymko et al. 2018; Burley et al. 2022). The non-invasive nature of many of these techniques, alongside improvements in affordability and portability, has allowed the development of a better understanding of the gross adaptative responses within the vasculature, across a range of populations and intervention strategies.

To date, the majority of research has focused on arterial responses to interventions, either in the periphery or cerebrovasculature, and changes in functional outcomes relating to arterial adaptation (e.g., FMD and cerebrovascular reactivity). However, there is growing interest in examining vascular responses outside of these regions, including within the microvascular of the coronary and cutaneous circulation. While acute flow responses are difficult to determine in humans, increases in the size and vasodilatory capacity of the coronary arteries are seen following exercise training (Laughlin et al. 2012). Within the cutaneous circulation in humans, exercise and passive heating stimuli significantly increased perfusion rate/vascular conductance, as assessed by Laser Doppler (Crandall and Wilson 2015; Green et al. 2017), alongside improvements in microvascular nitric oxide-mediated vasodilation following chronic interventions (Brunt et al. 2016a; Green et al. 2017). In addition to improving current understanding within specific vascular beds, greater breadth is needed in the range of vascular beds studied within intervention-based research. This is particularly important given the capacity for interventions to alter the distribution of blood flow across the vasculature. The focus of the current review is on the flow changes typically seen in the peripheral and cerebral circulation, as this is where the greatest level of understanding of acute and chronic flow responses is seen in vivo. However, future research must look to better characterise flow responses outside of these regions and to establish the link between these responses and the potential in vitro mechanisms driving them.

While we can characterise real-time flow responses in vivo within a particular vessel, how interventions may alter vascular function at the cellular level is not well understood. This is particularly important in relation to the endothelium, which is pivotal in driving vascular responses to interventions, and plays a central role in responding to changes in both flow and the circulating environment (Cahill and Redmond 2016). This is made more complex by the non-uniform patterns seen in acute responses between interventions and in the impact that changes in protocol can have on flow profiles (Fig. 1). Understanding the cellular responses in both an acute and adaptive context is central to uncovering the relationship between acute changes in flow and subsequent improvements in vascular health. This knowledge can then be integrated into the discussion surrounding the capacity to predict adaptive responses to an intervention by assessing the acute vascular response during a single visit (Dawson et al. 2018). The nature of the vascular system makes studying cellular responses in vivo incredibly challenging and we must look into in vitro models of the endothelium and flow response to build a complete picture of how the vasculature is responding to flow altering interventions.

### Flow-independent adaptation in vivo

Acute changes in the circulating environment have been well documented within in vivo research in humans; however, relatively little research to date has identified and characterised the role of these changes in long-term adaptation. In an exercise context, the beneficial effects of changes in the circulating environment for intervention adaptation remain allusive. However, research has linked changes in circulating levels of oxidative stress with a lack of adaptive responses in high-intensity continuous exercise. Goto et al. (2003) showed that over 12 weeks of training improvements in NO-dependent vasodilation were seen at moderate (50% *V*O_2max_) but not at high intensities (75% of *V*O_2max_), while high-intensity exercise alone drove significant increases in markers of oxidative stress. The authors hypothesised that this elevation in oxidative stress may have resulted in an inhibition of vasodilatory mechanisms, which attenuated any adaptive benefits that could have been derived from the elevations in systemic perfusion seen at high intensities. Interestingly, the same effects are not seen in interval-based high-intensity exercise, which have been shown to drive greater vascular adaptive responses than traditional steady-state moderate-intensity exercise (Ramírez-Vélez et al. 2019), potentially because of a reduction, rather than elevation, in oxidative stress during acute bouts (Bogdanis et al. 2013). This dynamic relationship between circulating markers of oxidative stress and subsequent adaptation to exercise demonstrates the potential for the circulating environment to have a critical impact on the efficacy of interventions. Whether the circulating environment plays broader roles across the vasculature remains to be seen. However, it could potentially provide a mechanism for vascular adaptation in regions that see reductions in blood flow during interventions that drive increases in vascular markers, as is seen in the cerebrovasculature during sprint-interval exercise (Weaver et al. 2021).

Within the peripheral vasculature, research has explored whether the occlusion of flow during repeated exposure to interventions alters adaptive responses, an approach that does provide some insight into the potential impact of circulating environment changes. To date, the majority of these studies have shown a loss of peripheral adaptation (e.g., improvement in FMD) within the occluded limb, leading to the hypothesis that vascular adaptations to interventions may be dependent on transient changes in flow/perfusion (Green et al. 2017). These studies include limb occlusion during exercise training (Birk et al. 2012) and heat-based interventions (Tinken et al. 2010), which also suggests that regardless of the intervention choice, adaptive responses may require alterations in flow. If correct, then it may be that vascular adaptation require changes in flow and that circulating responses provide additional stimuli, but are not sufficient in isolation. However, there are a number of caveats to these findings and the broader concept. First, the use of limb occlusion restricts the conclusions that can be drawn to the peripheral vasculature alone, particularly given that to date this hypothesis has primarily been explored in relation to the effects of occlusion on conduit artery function through measures of FMD. As such, it is possible that adaptations elsewhere in the vasculature are less dependent on changes in flow and more sensitive to changes in circulating environment. Second, the occlusion of flow may act to alter the exposure of the peripheral vasculature to changes in circulating environment. Even if systemic changes in the circulating environment are not altered, the reduction in flow to the limb will reduce the relative dose during the occlusion period. Given that in vivo it is difficult to determine the crucial time window for adaptive signalling, one potential reason for the complete loss of adaptive responses within the occluded limb could be that transient changes in the circulating environment during an intervention bout are central to cumulative adaptation over the course of an intervention. While there are methods available that could allow for exploration of flow-independent responses within other vascular beds and without the use of occlusion, such as end-tidal clamping of CO_2_ within the cerebrovasculature to limit elevations in flow (Prodel et al. 2016) or the use of nitric oxide synthase inhibitors to systemically limit flow elevations, the number of approaches are limited in vivo and have the potential to impact circulating environment changes or the downstream signalling pathways involved in response to vasoactive factors.

Given the complexity of examining the role of circulating factors in isolation in vivo, there is a clear need for better models to be developed in vitro that may allow for researchers to identify key markers for intervention strategies that could provide vascular benefits. To do so, we must first understand how individual factors affect the endothelium and how in concert they may alter cell signalling to drive functional adaptations. In doing so, we can explore the commonality between different intervention strategies and the potential cross-over and interaction between the circulating environment and changes in flow. From this understanding, future research can look to begin building more physiologically relevant in vitro conditions, providing a means for high-throughput modelling across different interventions and tissues without relying on animal- or disease-based models.

## Mechanisms driving vascular adaptation

### Flow-dependent mechanisms

#### Flow detection and response in endothelial cells

The vascular endothelium plays a central role in vessel function as the interface between the vessel and circulating blood, exemplified by the capacity for endothelial cells to respond to changes in blood flow. Early research during the 1980s identified the essential function of the endothelium in driving vasodilation, initially through endothelial ablation studies within canines (Pohl et al. 1986). Concurrently, in vitro research began to identify the functional components of the endothelium that drive relaxation of the smooth muscle cell layer and subsequent vasodilation. This included the identification and functional relevance of key vasoactive compounds, including nitric oxide (NO) (Palmer et al. 1987), prostacyclin (PGI_2_) (Moncada et al. 1976), and endothelin-1 (Yanagisawa et al. 1988). Alongside these vasoactive compounds, the endothelium was also shown to respond to shear stress through the opening of ion channels and subsequent hyperpolarization (Olesen et al. 1988), a key component in endothelium-dependent hyperpolarization, leading to subsequent hyperpolarization and relaxation of smooth muscle (Garland and Dora 2017). Subsequent research has identified a highly complex system of mechanosensitive proteins within the endothelium, that can detect the haemodynamic forces of shear stress and cyclic strain, and modulate responses to flow through mechanotransduction pathways in a force-dependent manner.

**Fig. 2:**
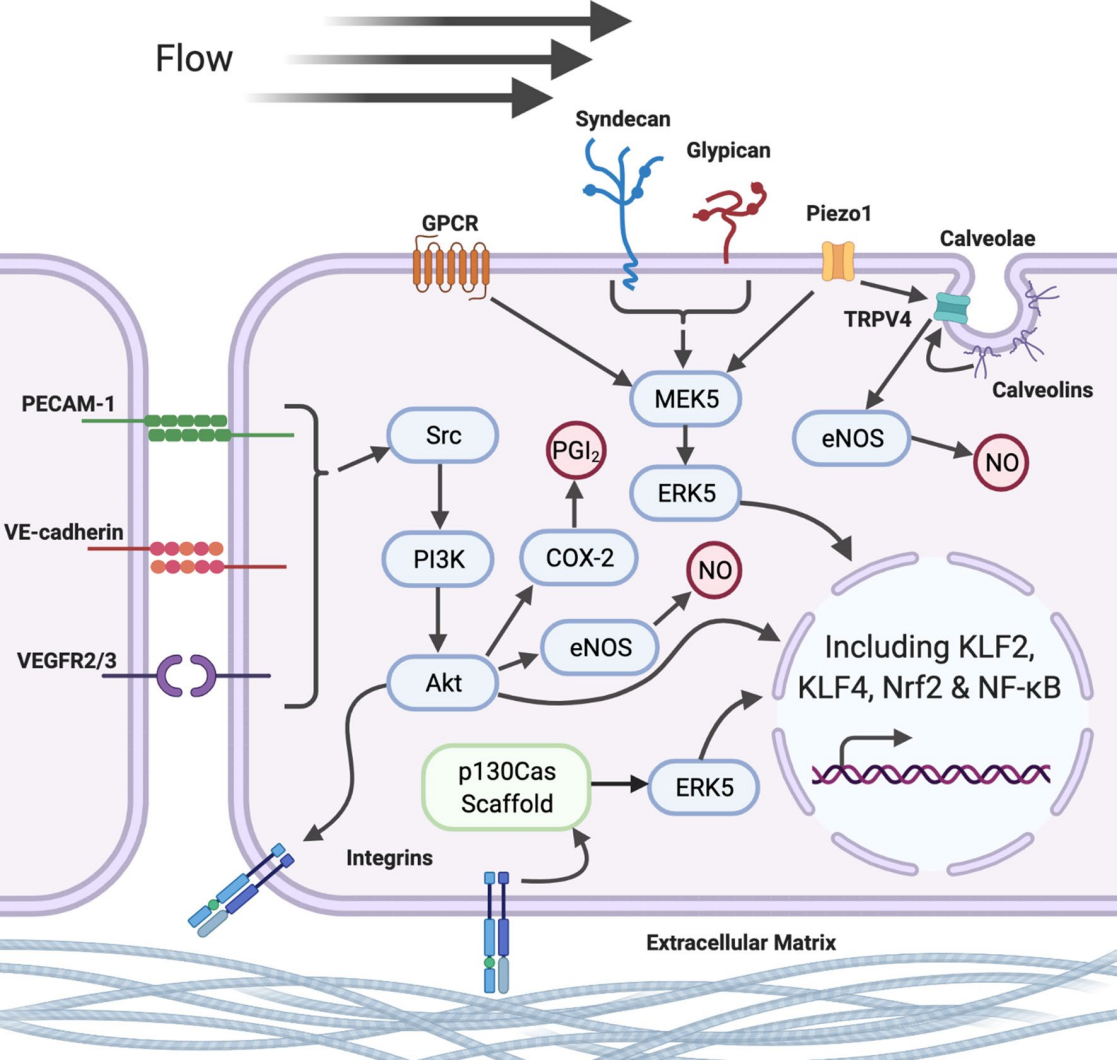
Flow-responsive mechanosensory pathways. Changes in flow are detected within the endothelial cell through several mechanosensory pathways. There are broadly four key signalling cascades regulating gene expression. Membrane bound mechanosensory receptors, including G-protein coupled receptors (GPCR), Piezo1 and transient receptor potential vanilloid-type 4 (TRPV4) ion channels and glycocalyx members (including syndecan and glypican), induce changes in gene expression via the mitogen-activated protein kinase pathway (MEK5/ERK5). In addition, the VE-cadherin–PECAM-1–VEGFR2/3 mechanosensory complex [comprising of VE-cadherin, platelet endothelial cell adhesion molecule 1 (PECAM-1), and vascular endothelial growth factor receptor 2 and 3 (VEGFR2/3)] can induce a cascade of responses, through the activation of Src-dependent phosphatidylinositol-4,5-bisphosphate 3-kinase (PI3K) and subsequent activation of the protein kinase Akt. Integrin-associated pathways can also induce signalling cascade responses via the p130Cas Scaffold and ERK5, either in response to activation via Akt signalling or through detection in changes in extracellular matrix structures in response to flow. Finally, activation of calveolins within membrane calveolae is also capable of inducing vasodilatory responses, via the activation of membrane-bound calcium (Ca^2+^) channels. Cumulatively, these signalling cascades induce changes in gene expression and acute vasodilatory responses via elevation in nitric oxide (NO) and prostacyclin (PGI_2_) production. Created with BioRender.com

Among the first proteins identified to be playing a role as mechanosensors were members of the glycocalyx glycoprotein layer that lines the internal surface of vessels at the interface between the blood and endothelial cell layer. These proteins were shown to respond to flow through the transduction of force to the cell membrane via glypican coreceptors or to the cytoskeleton via syndecan coreceptors (Baratchi et al. 2017). Numerous further mechanosensory proteins have since been shown to demonstrate an essential role in mechanotransduction, both in the detection of forces exerted on the cell and in the transduction of force from force sensors such as those within the glycocalyx. These include the VE-cadherin, platelet endothelial cell adhesion molecule 1 (PECAM-1) and vascular endothelial growth factor receptor 2 and 3 (VEGFR2/3) (VE-cadherin–PECAM-1–VEGFR2/3) mechanosensory complex (Tzima et al. 2005), caveolar proteins (Ariotti and Parton 2013), mechanosensitive ion channels (Baratchi et al. 2017), integrins (Janoštiak et al. 2014), and extracellular matrix component proteins (Fig. 2) (Baratchi et al. 2017).

While blood flow in vivo produces simultaneous shear stress and cyclic strain forces within blood vessels, these two forces are often addressed separately within in vitro research. Much of the research investigating the endothelial responses to mechanical force has been conducted using shear stress alone, due to the greater ease with which shear stress can be manipulated by the exposure of endothelial cells grown on a non-flexible surface to media under flow. As depicted in Fig. 2, at the onset of shear stress, the mechanical force exerted on the glycocalyx layer and other luminal mechanosensors activates the mitogen-activated protein kinase (MAPK) extracellular signal-related kinase 5 (ERK5), via MAP/ERK kinase 5 (MEK5) (Le et al. 2017). Simultaneously, elevated flow appears to drive shear-related responses within calveolae located within the endothelial membrane, through the activation of calveolin proteins, in particular calveolin-1 (Cav-1) (Sowa 2012). Both of these pathways have also been shown to play a key role in regulating responses in mechanosensitive ion channels and as such the potential for shear stress to drive calcium-dependent endothelial hyperpolarisation and subsequent activation of vasodilatory signalling mechanisms (Baratchi et al. 2017; Behringer 2017). Among these mechanosensitive ion channels, the transient receptor potential cation channel V4 (TRPV4) and Piezo1 have been identified as key mechanosensors and form the simplest mechanism for force-detection within the endothelium. Both Piezo1 and TRPV4 have been shown to increase endothelial permeability upon initiation of shear stress and appear to both play an essential role in subsequent calcium influx (Gerhold and Schwartz 2016). Recent research has also shown that TRPV4 is activated following the activation of Piezo1 (Swain and Liddle 2021) and is enhanced by activation and co-localisation with Cav-1 (Lu et al. 2017), suggesting that TRPV4 may play a central role in calcium-dependent flow responses.

Alongside the activation of luminal membrane proteins, flow-induced shear stress has also been shown to drive endothelial responses through the activation of adhesion molecules involved in cell–cell and cell–extracellular matrix (ECM) interactions. The most well studied of these pathways is the VE-cadherin–PECAM-1–VEGFR2/3 mechanosensory complex comprising of VE-cadherin, platelet endothelial cell adhesion molecule 1 (PECAM-1), and vascular endothelial growth factor receptor 2 and 3 (VEGFR2/3), which was first identified by Tzima et al. (2005). In response to increased shear, VE-cadherin transfers tension to PECAM-1 and facilitates the association between PECAM-1 and VEGFR2/3 (Conway et al. 2013). This leads to the activation of signalling cascades through phosphoinositide 3-kinase (PI3K) (Jin et al. 2003), integrin-associated pathways (Hahn and Schwartz 2009), and subsequently altering gene expression (Fig. 2; see “Flow-independent adaptation in vivo”). The essential role that this complex plays in mechanotransduction was demonstrated initially in bovine aortic endothelial cells (BAEC); in which loss of VE-cadherin or PECAM-1 resulted in loss of PI3K responses (Tzima et al. 2005). Subsequently, similar PI3K responses under flow have also been seen in human umbilical vein endothelial cells (HUVEC) under the inhibition of all three components within the VE-cadherin–PECAM-1–VEGFR2/3 complex (Jin et al. 2003; Coon et al. 2015; Russell-Puleri et al. 2017).

Evidence also suggests that shear stress can be detected through conformational changes in the extracellular matrix and ECM–cell interactions. However, this is less well understood, in large part due to the high level of complexity and regional specificity (Wheeler et al. 2014) that likely enables highly specific responses in different vascular beds (Baratchi et al. 2017). Although much research is still needed to understand the scope and role of the ECM in flow responses, integrins appear to play a pivotal role in this mechanosensory mechanism. Specifically, integrins mediate interactions between the ECM and mechanosensory properties of PECAM-1 (Collins et al. 2014), and enable ECM-dependent mechanotransduction and subsequent signalling pathways via the p130Cas scaffold complex (Janoštiak et al. 2014). First identified by Yamada et al. (2006), the p130Cas scaffold complex has been shown to associate with cell membrane integrins and other focal adhesions (Defilippi et al. 2006; Baratchi et al. 2017), through which changes in ECM conformation can drive phosphorylation of the CAS substrate domain and the induction of ERK and other signalling pathways (reviewed in Janoštiak et al. 2014), and subsequent changes in gene expression (see “Flow-independent adaptation in vivo”). This unique mechanosensory pathway has the potential to alter flow-dependent responses across different regions, due to the complexity and variability in ECM composition across the vasculature (Collins et al. 2014), and warrants further investigation, particularly when looking to understand flow responses within a specific vascular bed.

As mentioned, the majority of our understanding surrounding these mechanosensory pathways come from the application of shear stress. However, growing evidence indicates that cyclic strain has a unique impact on endothelial responses to mechanical force (Meza et al. 2019). Cyclic strain in isolation has been shown to activate a number of mechanosensory pathways, through stretch-activated ion channels, ECM–cell adhesions (via the activation of integrins), and cell–cell interactions [via the VE-cadherin–PECAM-1–VEGFR2/3 mechanosensory complex (Jufri et al. 2015)]. Collectively, these mechanisms enable cyclic strain to induce significant signalling responses and have been shown to induce changes in cell morphology and orientation in the absence of shear stress (Bernardi et al. 2018). When investigated in combination with shear stress, the two haemodynamic forces have an additive effect on mechanosensory signalling pathways, although this response is not fully synergistic in nature, most likely due to the overlap in the pathways responsible for detecting shear and strain (Meza et al. 2019). Furthermore, the interaction between these forces becomes more complex when considering the relative strength and dynamics of each force and the structural variability seen across the vasculature (Gray and Stroka 2017).

#### Signal cascade responses to flow exposure

**Fig. 3:**
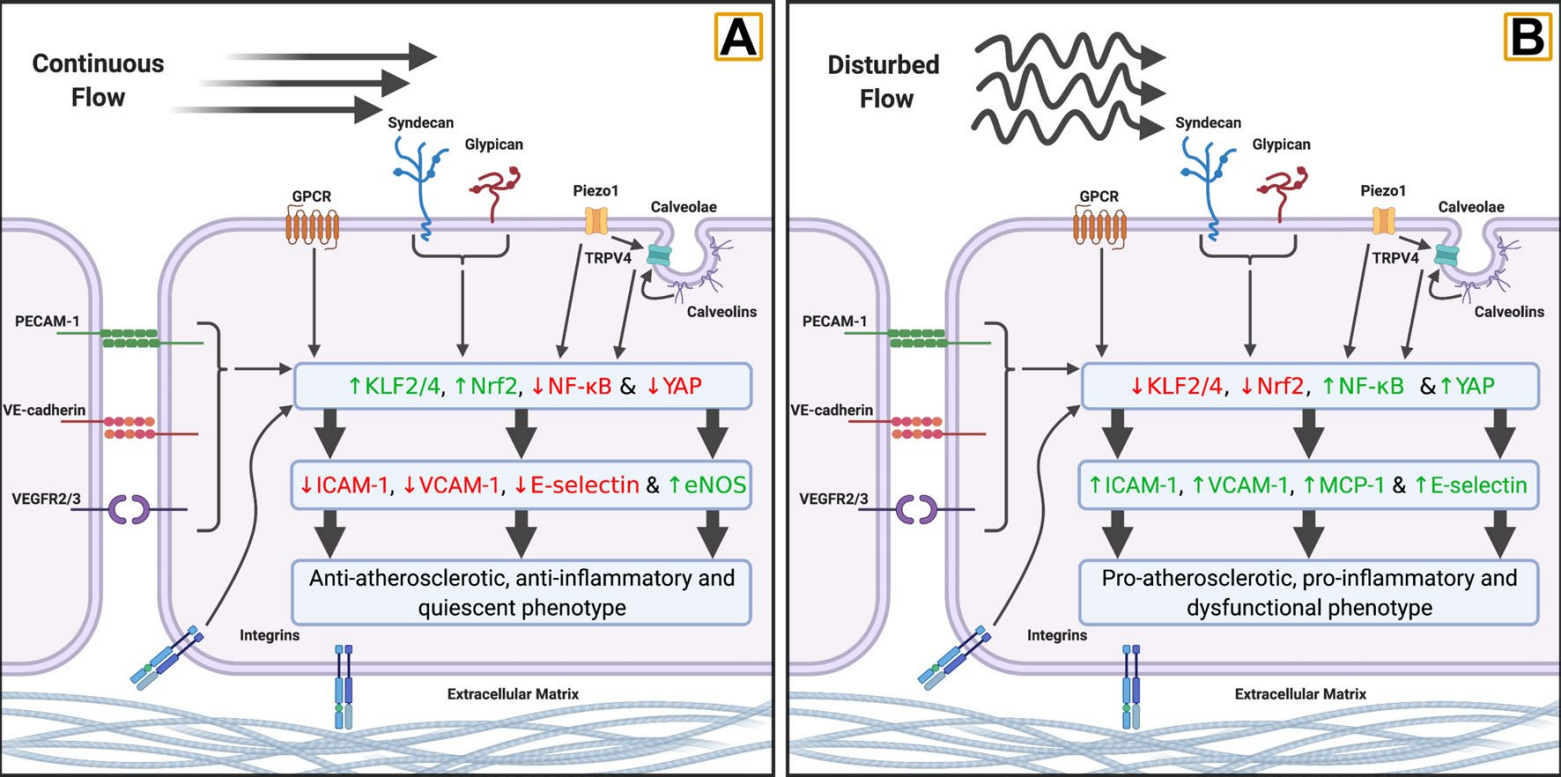
Differences in typical response between continuous and disturbed flow models. **a** Prolonged continuous and elevated flow results in the promotion of a quiescent endothelial cell phenotype through the upregulation of Krüppel-like factor 2 and 4 (KLF2/4) and Nuclear-factor-E2-related factor 2 (Nrf2), while suppressing the activity of Nuclear-factor kappa-B (NF-κB) and Yes-associated protein 1 (YAP), via the array of mechanosensory pathways detailed in Fig. 2. **b** Disturbed flow, characterised by oscillatory patterns of low or reversing shear stress, activates mechanosensory pathways through many of the same receptors as seen in continuous flow; however, these patterns drive opposing responses to those seen in continuous flow leading to the promotion of a dysfunctional endothelial phenotype. Created in BioRender.com

The endothelial response to changes in blood flow, and the associated mechanosensory response to changes in shear stress and cyclic strain, is highly dependent on the nature of the flow change itself (i.e., the pattern of flow) both in terms of acute signalling responses and subsequent adaptive changes. While the mechanosensory pathways are activated under different flow profiles and the nature of the relationship between different sensors is not well understood, clear differences are seen in subsequent signal pathway responses between flow patterns (Fig. 3) (Nakajima and Mochizuki 2017). In vitro modelling has been used to identify the nature of these differences, including mimicking in vivo disease states; such as ischemic reperfusion injury, or structural variations that can alter regional flow in vivo; such as stenoses, bifurcations or arched structures (e.g., aortic arch) (Gray and Stroka 2017).

**Fig. 4:**
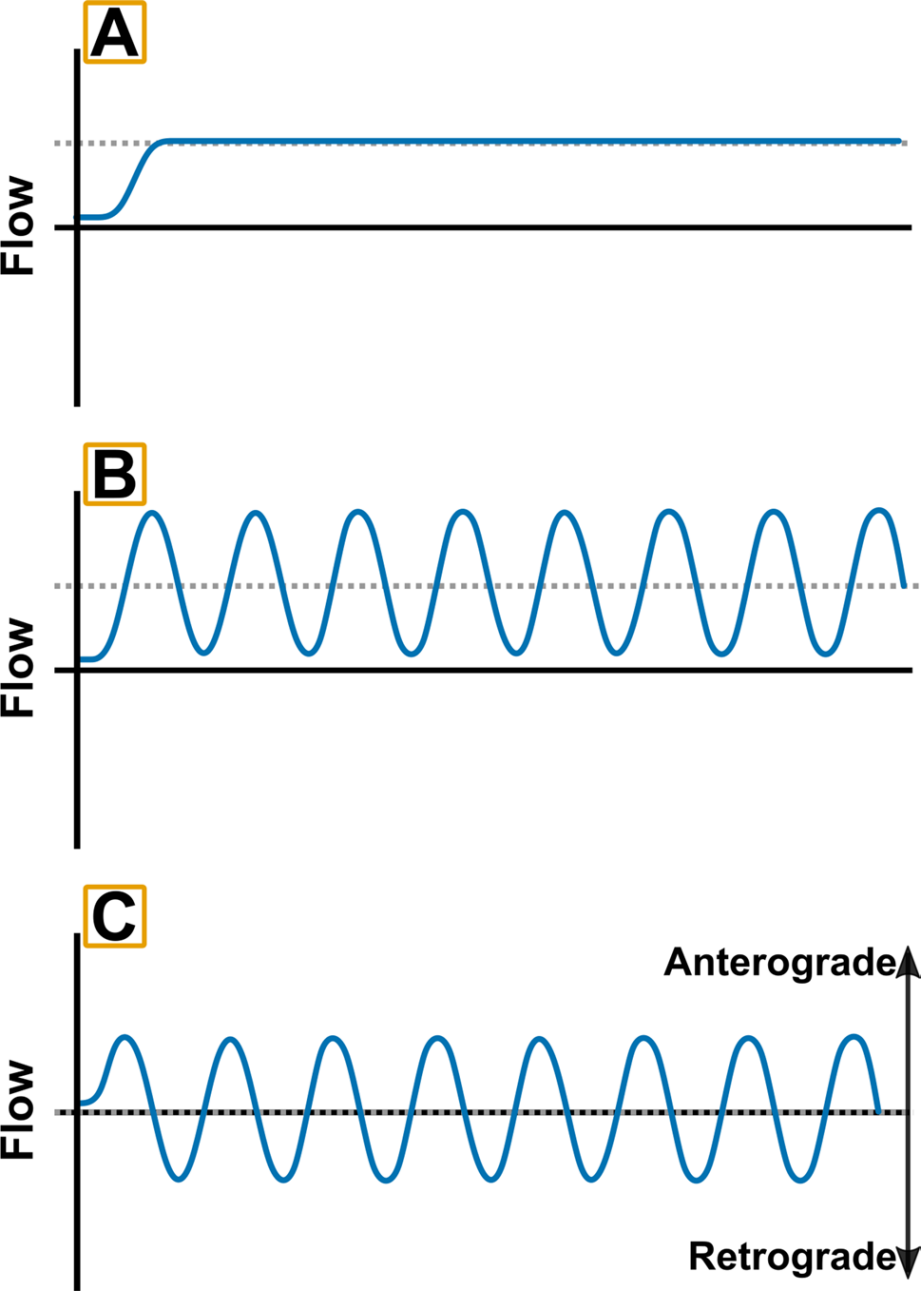
Flow patterns within continuous and disturbed cell culture conditions. Illustration of simple flow patterns utilised to induce continuous and disturbed responses within endothelial cells. Mean flow rate induced by each pattern is shown as a dashed line in all plots. Continuous, elevated flow responses can be induced by the application of laminar (**a**) or pulsatile (**b**) flow patterns in cell culture. Pulsatile flow can also be utilised to simulate disturbed conditions, by the application of pulsatile profiles at low average flow rate (**c**), resulting in anterograde and retrograde flow components

Distinct endothelial phenotypes are seen across the vasculature, as a result of regional and tissue-specific variability in both local microenvironment and the function of the endothelium within different tissues (reviewed in Aird 2007a, b). Broadly speaking this heterogeneity has been observed in endothelial cells from different vascular beds (e.g., arteries vs. veins; Aird 2007b); across different specialised tissue beds (heart, lungs, kidneys, liver, and brain) (Cleuren et al. 2019; Jambusaria et al. 2020); and even in different regions within the same vessels (Simmons et al. 2012). Crucially the plasticity of the endothelium to adapt to changes in stimuli can result in the rapid loss of the in vivo phenotype when the cells are cultured in vitro (Aird 2007b; Kuosmanen et al. 2017). This plasticity can be beneficial in developing cell culture models, particularly where cells from relatively accessible sources, such as HUVEC isolated from umbilical cord, can be manipulated to express phenotypes akin to those found in less-accessible vascular beds. This is seen in HUVEC-based models of arterial flow, where arterial levels of shear stress (approx. 2.0 Pa or 20 dyn/cm^3^), of either a constant or pulsatile nature (Fig. 4), induce a well-characterised pattern of response (discussed below) comparable to those seen in BAEC (Conway et al. 2013) and other arterial cell types (Luu et al. 2010). Conversely, while plasticity enhances our ability to induce a particular phenotype through the manipulation of culture conditions, it also makes the maintenance of in vivo phenotypes in isolated cells or the modelling of specific vascular beds more complicated (Aird 2007b). For example, in modelling of the blood–brain barrier, researchers must consider the selection of appropriate endothelial cell sources; the essential role of astrocytes, pericytes, and other cells of the neurovascular unit; alongside appropriate simulation of the microenvironment on both sides of the barrier (Sivandzade and Cucullo 2018). To date, the majority of studies investigating flow and intervention responses have focused on a relatively small pool of endothelial cell types and generally under linear, arterial flow. While this is in keeping with the majority of human in vivo research, future considerations (see “Mechanisms driving vascular adaptation”) must look to expand the field in keeping with the advancement in technology facilitating the measurement of flow outside of conduit arteries (Baratchi et al. 2017).

Continuous elevation of flow induces rapid responses in the expression of key pro-quiescent transcription factors (Fig. 3a), most prominently krüppel-like factor 2 and 4 (KLF2 and KLF4) and nuclear-factor E2-related factor 2 (Nrf2) via the ERK5 signalling pathway (Fledderus et al. 2008). While elevation in the activity of nuclear-factor kappa-B (NF-κB) and the flow-responsive transcriptional co-regulator YAP, key factors in pro-inflammatory pathways, are also seen under continuous flow, this is highly transient and is followed by rapid reduction in activity over time (Nakajima and Mochizuki 2017). Combined with the rapid activation of p130Cas signalling pathways through the VE-cadherin–PECAM-1–VEGFR2/3 complex and ECM–cell-associated integrins, KLF2 mediates changes in vascular tone through the upregulation of endothelial nitric oxide (NO) synthase (eNOS) (Baratchi et al. 2017). Simultaneously, these signalling responses have been linked to upregulation of anti-inflammatory factors; including eNOS and thrombomodulin (Lin et al. 2005); suppression of inflammatory molecules; including intercellular cell adhesion molecule 1 (ICAM-1), VCAM-1 and E-selectin (Baratchi et al. 2017; Ajami et al. 2017); and improvements in redox balance via the Nrf2-antioxidant response (Fledderus et al. 2008).

Contrastingly, oscillatory or disturbed flow patterns (exemplified by repetitive changes from high-to-low flow or anterograde-retrograde flow, Fig. 4) drive relatively small responses in KLF2/4 and Nrf2. Disturbed flow patterns trigger significant elevations in pro-atherosclerotic and pro-inflammatory signalling pathways (Fig. 3b); most prominently via Nf-κB and YAP (Nakajima and Mochizuki 2017). Sustained elevation in both factors under disturbed flow leads to a significantly more-pronounced inflammatory response (Nakajima et al. 2017); including increases in ICAM-1, VCAM-1, E-selectin, and Monocyte Chemoattractant Protein-1 (MCP-1) (Chiu and Chien 2011). This difference in signalling response is driven by differences in mechanosensory activity between laminar and disturbed flow, demonstrated in the contrasting role of PECAM-1 between the two conditions. Under elevated laminar flow, acute increases in PECAM-1 signalling are essential for both acute shear-dependent vasodilatory responses (Bagi et al. 2005; Russell-Puleri et al. 2017) and subsequent vascular remodelling under exposure to chronic stimuli (Tzima et al. 2005). While under pathological and disturbed flow, PECAM-1 plays an essential role in NF-κB activation and downstream over-expression of adhesion molecules (Tzima et al. 2005). Furthermore, in PECAM-1 knock-out mice, disturbed flow has been linked to reductions in vessel diameter (Chen et al. 2010) and atherosclerosis severity (Harry et al. 2008). In combination, the changes induced by prolonged disturbed flow lead to the establishment of a dysfunctional, pro-atherosclerotic endothelium exhibiting elevated levels of inflammation, oxidative stress, and vasoconstriction (Chiu and Chien 2011). While this phenotype is likely typical under chronic, pathological conditions, such as atherosclerosis, a lack of current research makes understanding the impact of shorter periods of oscillatory flow difficult. Given the positive impact that pneumatic compression can have on vasodilatory function, it is likely that shorter, transient periods of flow of this nature have a different impact on endothelial response. This makes comparison between in vitro research and the potential mechanisms driving responses to intervention difficult (Table 1) and warrants further research into the impact of transient exposure to oscillatory flow.

**Table 1:** Comparison of in vitro endothelial cell and in vivo vascular adaptive responses to common stimuli found during non-pharmacological interventions

Stimulus	Interventions	In vitro	In vivo
Continuous elevation in flow	Exercise Water immersion Heat therapy	↑ eNOS ↓ Inflammatory cytokines ↑ Antioxidants ↑ Anti-inflammatory molecules	↑ Basal blood flow ↑ Maximal flow-mediated dilation ↑ Cerebrovascular responsiveness
Intermittent elevation in flow	Exercise Heat therapy	???	???
Oscillatory flow	Pneumatic compression	*Prolonged exposure:* ↑ Inflammatory cytokines ↑ Adhesion molecules ↑ Vasoconstriction factors	↑ Basal blood flow Improved functional outcomes
Loss of flow	Ischemic preconditioning	*Prolonged exposure:* ↑ Inflammatory cytokines ↑ Cellular dysfunction ↑ Cell death	↑ Endothelium-dependent blood flow ↑ Maximal flow-mediated dilation
Hypoxia	Ischemic preconditioning Hypoxia	↑ Inflammatory cytokines ↑ Oxidative stress ↑ Angiogenic factors ↑ eNOS	???
Increased temperature (Febrile/ ~ 39 °C)	Heat therapy	↓ IL-6 response to TNFa ↓ Future hyperthermic damage HSP protection against inflammation	↑ Basal blood flow ↑ Maximal flow-mediated dilation ↑ Cerebrovascular conductance

References for the stated responses can be found in Sects. “Ischemic preconditioning”, “Flow-independent adaptation in vivo” and 3.3

*eNOS* endothelial nitric oxide synthase, *IL* interleukin, *VEGF* vascular endothelial growth factor, *heat shock protein 72* HSP72, *tumor necrosis factor alpha* TNFα, ↑ increase, ↓ decrease,??? response currently unknown

Less well-studied in vitro is the endothelial response to changes in flow pattern in cultures grown under flow conditions and how short, transient changes may lead to subsequent adaptive changes in endothelial function. These transient changes in flow are a key focal point within in vivo human research and a central tenant of the mechanisms driving adaptations to a range of interventions including exercise (Green et al. 2017), passive heating (Cheng et al. 2019), and pneumatic compression (Credeur et al. 2019) (see “Ischemic preconditioning”). While limited research has been conducted to date, in vitro research has shown the potential for acute changes in flow to stimulate beneficial signalling pathways within the endothelium. In endothelial cells preconditioned to arterial levels of shear stress, further elevations in shear have been shown to stimulate increases in the expression of a range of genes including KLF2 and KLF4, alongside the upregulation of eNOS activity and the promotion of an anti-inflammatory and atheroprotective phenotype (Zhang and Friedman 2012). Furthermore, elevation in the frequency of pulsatile flow, as would be seen during interventions with the capacity to elevate heart rate, leads to increases in the expression of angiogenic and cell proliferation genes, as well as upregulation of VEGF (Zhang and Friedman 2013). These studies demonstrate that elevations or changes in flow conditions can drive significant mechanosensitive response, whether in cells previously cultured under static or continuous flow conditions. Additionally, they provide a potential mechanism for the vascular changes seen under interventions that drive continuous flow elevations (Table 1), and align with the improvements in NO-mediated vasodilatory function that are seen following interventions such as passive heating and exercise (Green et al. 2017; Cheng et al. 2019). The capacity for increases in both shear rate and pulsatile frequency to induce changes in endothelial responses also demonstrates the complex nature of flow responses within the endothelium and the need for specific models of intervention responses. Future research must also consider the impact of the duration of flow changes on subsequent responses, as most interventions induce changes in the magnitude and frequency of pulsatile flow, over a relatively short time-course and often in a non-continuous manner (see Fig. 1).

While most prominently studied from the perspective of disease, research has been conducted into the impact that reduction or loss of flow during ischemic reperfusion can have on previously perfused cells in vitro. Under ischemic reperfusion, the endothelium sees a rapid elevation in ROS and suppression of vasodilation via eNOS uncoupling, coupled with a transition into a more pro-thrombotic and pro-inflammatory state, via the upregulation of hypoxia-inducible factor 1 (HIF-1α) and NF-κΒ (Hernández-Reséndiz et al. 2018). While ischemic events in vivo consist of both a loss or significant reduction of flow and exposure to a hypoxic environment, cessation of flow alone has been shown to drive significant signalling responses including the activation of NADPH oxidase 2 (NOX2) and subsequent production of ROS, the induction of pro-inflammatory transcription factors (including HIF-1α and NF-κΒ) (Chatterjee 2018) and the downregulation of KLF2 (Matharu et al. 2008). Although prolonged, uncontrolled periods of ischemia can have severe pathological complications, including cellular dysfunction, cardiovascular complications and death, shorter controlled periods of ischemia in the form of ischemic preconditioning are thought to elicit beneficial adaptations. These adaptations include improving vascular function within both the periphery and cardiac circulation and improving outcomes in future ischemic reperfusion injury (Tapuria et al. 2008). It has been suggested that these benefits result from the lower level of cellular distress caused by short bouts of ischemia, which are insufficient to impact on cell viability and death, but capable of driving adaptive responses to condition the endothelium against the potential damage of further ischemic events (Kalogeris et al. 2012; Hernández-Reséndiz et al. 2018). This is seen in the time-course of the inflammatory responses seen following cessation of flow; as assessed by neutrophil adherence under TNFα exposure, in which relatively little change is seen over the first 30 min of flow cessation (Matharu et al. 2008). However, further research is needed to formally address endothelial responses to brief periods of ischemia, as used in IPC interventions, as well as in relation to hypoxia and loss of flow both separately and in combination.

### Flow-independent mechanisms

#### Signal cascade responses to changes in culture conditions

Alongside the impact that environmental manipulation (e.g., hypoxic conditioning or passive heating) can have on blood flow (see “Ischemic preconditioning”), environment-based interventions also induce significant changes in the internal environment within blood vessels and the endothelium (e.g., temperature, oxygen tension/availability). In the case of ischemic preconditioning, much of our understanding regarding the impact that changes in temperature or oxygen availability have on the endothelium comes from research focused on pathological conditions. Interestingly, in vitro endothelial cells respond to hypoxia by elevating signalling via NF-κB and HIF-1α (D’Ignazio and Rocha 2016) in a similar manner to the responses seen under ischemic conditions (Hernández-Reséndiz et al. 2018), suggesting similar mechanisms of response whether oxygen is reduced via ischemia or environmental manipulation. In vivo research has demonstrated clear differences between moderate (9–16% inspired O_2_) and extreme (2–8% inspired O_2_) hypoxic “doses”; including beneficial vs. detrimental impact on blood pressure and systemic inflammation (Navarrete-Opazo and Mitchell 2014), akin to the differences seen between transient and sustained ischemic reperfusion (Kalogeris et al. 2012). In vitro, similar differences were seen when HUVEC were incubated at 0, 5, and 10% O_2_, across 6-, 12-, and 24-h periods, with elevations in HIF-1α and NK-κB, subsequent increases in ICAM-1, VEGF and reductions in cell viability occurring in a time- and O_2_ concentration-dependent manner (Baldea et al. 2018). While this study shows the potential impact of dose and exposure duration, the majority of in vitro studies have focused on extreme hypoxia reflective of pathological conditions, and more refined, dose-dependent response experiments are needed to understand the potential beneficial and detrimental effects of these differences at the cellular level (Pavlacky and Polak 2020).

Similarly, heat stress can alter endothelial signalling pathways and drive adaptive responses beyond those induced by changes in flow alone. The impact of hyperthermia on endothelial cells appears to be highly dependent on the temperature and conditions of the exposure. In addition to increases in eNOS expression and NO release (Harris et al. 2003), severe hyperthermia (≥ 41 °C) also triggers upregulation in adhesion molecules such as ICAM-1 (Lefor et al. 1994), and induction of endothelial apoptosis via a p53-dependent mechanism (Gu et al. 2014). This response appears to depend on the severity of the hyperthermic stimulus, as febrile temperatures (38–40 °C) drive little basal response in inflammatory cytokines (including IL-1Β, IL-6 and IL-8) or adhesion molecules (including ICAM-1, VCAM-1 and PECAM-1) (Shah et al. 2002). This difference in basal response may also relate to the anti-inflammatory effects febrile temperatures have on the endothelium, such as a reduction in TNFα-induced IL-6 secretion (Hasday et al. 2001). The duration of exposure to heat stress also appears to alter the severity and nature of endothelial responses, with little apoptotic cell death seen following 90 min at 42 °C (Liu et al. 2015) and a reduction in TNFα-induced cell adhesion molecule expression after 60-min at 42 °C compared to normothermic controls (Nakabe et al. 2007). NF-κB is thought to play a key role in regulating these responses to hyperthermia, both during exposure to heat stress and in the subsequent impact on endothelial function post-hyperthermia. Hyperthermia stimulates increases in NF-κB to protect against heat-induced apoptosis Liu et al. (2015), with NF-κB levels subsequently decreased following hypoxic-reoxygenation stimuli (Brunt et al. 2018) and were linked to reductions in adhesion molecule responses to TNFα treatment (Nakabe et al. 2007). It is possible that the divergent NF-kB responses to heat stress are analogous to those seen in response to changes in shear stress (see “Flow-independent adaptation in vivo”). Under this hypothesis, shorter or febrile temperature exposure may induce transient elevations in NF-κB that effectively “primes” the endothelium and promotes an anti-inflammatory response, while prolonged exposure to high temperatures leads to continuous elevation in NF-κB and promotion of a pro-inflammatory, apoptotic phenotype. Future research should examine this hypothesis and determine the role that magnitude and duration play in hyperthermic responses. This is particularly important if we wish to interpret the impact of heat-based interventions, as typically small changes in core temperature (~ 1–2 °C) are induced for short periods, while most in vitro research to date has focused on longer exposures above 40 °C.

To summarise, across the wide range of unique intervention strategies utilised for vascular health, clear similarities can be seen in the capacity for dose-dependent responses within the endothelium, whether the dose response is related to magnitude of flow change, duration of stimulus or the level of deviation from “normal”/ “healthy” conditions. These response patterns highlight the importance of specifically designing experiments to study the impact of interventions on the endothelium, rather than relying on the re-interpretation of findings from dysfunction or disease-based models.

#### Interaction with signalling factors, inflammatory cytokines, and the circulating environment

Adding further complexity to our understanding of interventions and their effects on the endothelium is the manner in which different strategies may alter the circulating environment; specifically in the form of changes in circulating signalling molecules and vasoactive substances occurring in vivo. These changes can be: (1) induced locally by the endothelium itself in response to changes in flow; particularly in relation to the release of vasoactive substances and inflammatory cytokines; (2) produced by other tissues and released into the circulation, or (3) induced in an endocrine manner within the endothelium as a positive feedback loop response to circulating signalling factors released from other tissues.

In vivo the endothelium is rarely impacted in isolation and more commonly changes in flow are driven alongside changes in both proximal and distal tissues, making the role of circulating signalling factors central to understanding the holistic impact of non-pharmacological interventions. The importance of changes in circulating factors is seen across a range of interventions and is best evidenced in exercise and passive heating, which despite their differences in aetiology (e.g., active recruitment of muscle contraction vs passive exposure to a heat stimulus), both produce marked elevations in core temperature and blood flow (Naylor et al. 2011; Francois and Thomas 2016). Despite the methodological contrast between these two approaches, similar responses are seen not only in haemodynamic responses (see “Ischemic preconditioning”), but also in vascular biomarkers including interleukin-6 (IL-6), VEGF, and HSP72 (Henstridge et al. 2016; Hoekstra et al. 2018; Akerman et al. 2019). The similarities in the known acute response to these two strategies have led to suggestions that they may induce similar vascular benefits to one another, as a result of comparable elevations in heart rate and increases in both core and skeletal muscle temperature during acute bouts (reviewed in Hoekstra et al. 2020). Interestingly, acute circulating factor responses are also seen during hypoxic stimuli (Table 2), including elevations in both HSP72 and VEGF (Taylor et al. 2010; Burki and Tetenta 2014). These complementary acute responses across the three intervention strategies are matched in their responses to repeated exposure, most notably in the reduction in circulating markers of inflammation across exercise training (Palmefors et al. 2014), hot water bathing (Hoekstra et al. 2020), and dose-dependently in intermittent hypoxia (Navarrete-Opazo and Mitchell 2014).

**Table 2:** Acute responses in circulating factors seen in exercise-, heat-, and hypoxia-based interventions in humans

Factor	Exercise	Heat	Hypoxia
IL-1Rα	Y^1^	Y^5^	Y (chronic)^8^
IL-1β	Y^1^	?	?
IL-6	Y^2^	Y^5,6^	Y(chronic)^8^
IL-8	Y^2^	Y^5^/N^7^	?
IL-10	Y^2^	?	?
VEGF	Y^3^	N^7^	Y^9^
HSP72	Y^4^	Y^6^	Y^10^
TNFα	Y^1^	N^5,7^	N^9^

Factors are marked dependent on whether an elevation (**Y**) or no change (**N**) is seen with a given intervention stimulus. If responses are only seen over chronic exposure (> 24-h) to a stimulus then this is also denoted (**chronic**). Factors are also marked if the response is not well understood (**?**)

*IL* interleukin, *VEGF* vascular endothelial growth factor, *heat shock protein 72* HSP72, *tumor necrosis factor alpha* TNFα

References: ^1^(Ostrowski et al. 1999); ^2^(Kasapis and Thompson 2005); ^3^(Landers-Ramos et al. 2014); ^4^(Henstridge et al. 2016); ^5^(Leicht et al. 2015); ^6^(Faulkner et al. 2017); ^7^(Kuhlenhoelter et al. 2016); ^8^(Hartmann et al. 2000); ^9^(Burki and Tetenta 2014); ^10^(Taylor et al. 2010)

One possible explanation for the similarities in both acute and chronic responses to a wide variety of intervention strategies is the central role that hypoxia-inducible factor 1α (HIF-1α) plays in angiogenesis and vascular function. In vivo human research has demonstrated that both acute and chronic exposure to heat or hypoxia results in similar elevations in extracellular HIF-1α (Lee et al. 2016), while elevations in skeletal muscle due to localised hypoxia during exercise have been suggested to play an essential role in adaptation to training (Lindholm and Rundqvist 2016). Although it is not possible to elucidate the impact that changes in HIF-1α have on the endothelium directly in vivo, HIF-1α plays a clear role in endothelial function in vitro. Indeed, the HIF-family of proteins have been highlighted as potential master regulators for angiogenesis during vascular development (Hashimoto and Shibasaki 2015), driving elevations in NO production via eNOS; the release of pro-angiogenic factors such as VEGF, and activation of inflammatory pathways via increased interleukin release (Krock et al. 2011). Research in rodent models has also shown that hypoxia-inducible factors are essential to the acute inflammatory response (including IL-4, IL-6, and IL-10) following ischemic preconditioning (Cai et al. 2013; Yang et al. 2018) and the subsequent beneficial signalling responses via Akt (Du et al. 2020), further demonstrates the central role that HIF signalling plays. The similarities and potential role of hypoxia-inducible factors across different intervention stimuli may be pivotal in the cross-over seen between many of the interventions discussed within this review, such as the cross-adaptation following heat acclimation interventions driving improvement in exercise tolerance in hot and hypoxic conditions (Lee et al. 2016; Gibson et al. 2017).

This is not to say that changes in the circulating environment are not highly dependent on the specific parameters of a given intervention, in much the same manner as is seen in the responses to flow between interventions. This is demonstrated in the variable responses to changing parameters within a single intervention type, such as the protocol-dependent responses in circulating IL-6, TNFα, VEGF, and other factors seen following exercise-based interventions (Palmefors et al. 2014; Ramos et al. 2015) and the temperature-dependent response in HSP70 seen following active heat acclimation (Lovell et al. 2007). The complexity of the circulating environment makes determining the full scope of a given intervention’s impact very difficult, let alone the potential variations in response within and between different intervention paradigms. This is particularly true in relation to the current approach taken to understanding the circulating environment. This is often done by targeting specific biomarkers of response (e.g., VEGF, HSP’s, HIF-1α, etc.), which by their very nature are likely to display similar responses to interventions that have been selected specifically for their ability to stimulate vascular responses. To address this dilemma, future research must look to expand the breadth of targets investigated within the circulation, as can be achieved through the utilisation of omics approaches that allow for the full scope of responses within the circulation to be determined (Hoffman 2017).

Our understanding of the impact that many circulating factors have on the endothelium at the cellular level suffers from a similar issue, in that the molecular response to many factors is well understood in isolation and in relation to pathological conditions. However, our knowledge of the cellular response to the collective changes seen during non-pharmacological interventions is much less developed. This is exemplified in our understanding of the impact that inflammatory cytokines have on the endothelium, which have been studied and reviewed at length in relation to vascular dysfunction and disease (Sprague and Khalil 2009). Elevated inflammatory cytokine levels have been shown to play a central role in endothelial dysfunction through disruption of vasodilatory mechanisms, sustained upregulation of angiogenic factors and activation of NF-κB signalling pathways (Sprague and Khalil 2009). Additionally, prolonged periods of cytokine elevation are thought to promote the development of hypertension (Pioli and de Faria 2019), atherosclerosis (Tedgui and Mallat 2006), and chronic vascular dysfunction (Steyers and Miller 2014). When we look to interpret the potential role that these mechanisms play within interventions, similarities can be drawn in the transient response to interventions such as exercise and passive heat exposure (Kasapis and Thompson 2005; Hoekstra et al. 2018); in which significant elevations in inflammatory cytokines likely elicit similar signalling responses to those seen in inflammatory disease or dysfunction. However, over repeated exposure to these strategies a reduction is seen in basal inflammatory cytokine profiles (Gleeson et al. 2011; Hoekstra et al. 2020), in stark contrast to the response seen in dysfunction. This is likely due to the transient nature of exposure during interventions compared to chronic elevations seen in pathological states. It has been suggested that this difference is key to the anti-inflammatory benefits of a number of interventions (Hoekstra et al. 2020), in a similar manner to the transient periods of reduced flow seen in ischemic preconditioning that drives beneficial adaptive responses in vascular function (Kalogeris et al. 2012; Hernández-Reséndiz et al. 2018). This also highlights the importance of considering differences in the nature, magnitude, and duration of changes in the circulating environment during interventions, especially when looking to compare the potential mechanisms driving endothelial responses from pathophysiological models and studies.

**Fig. 5:**
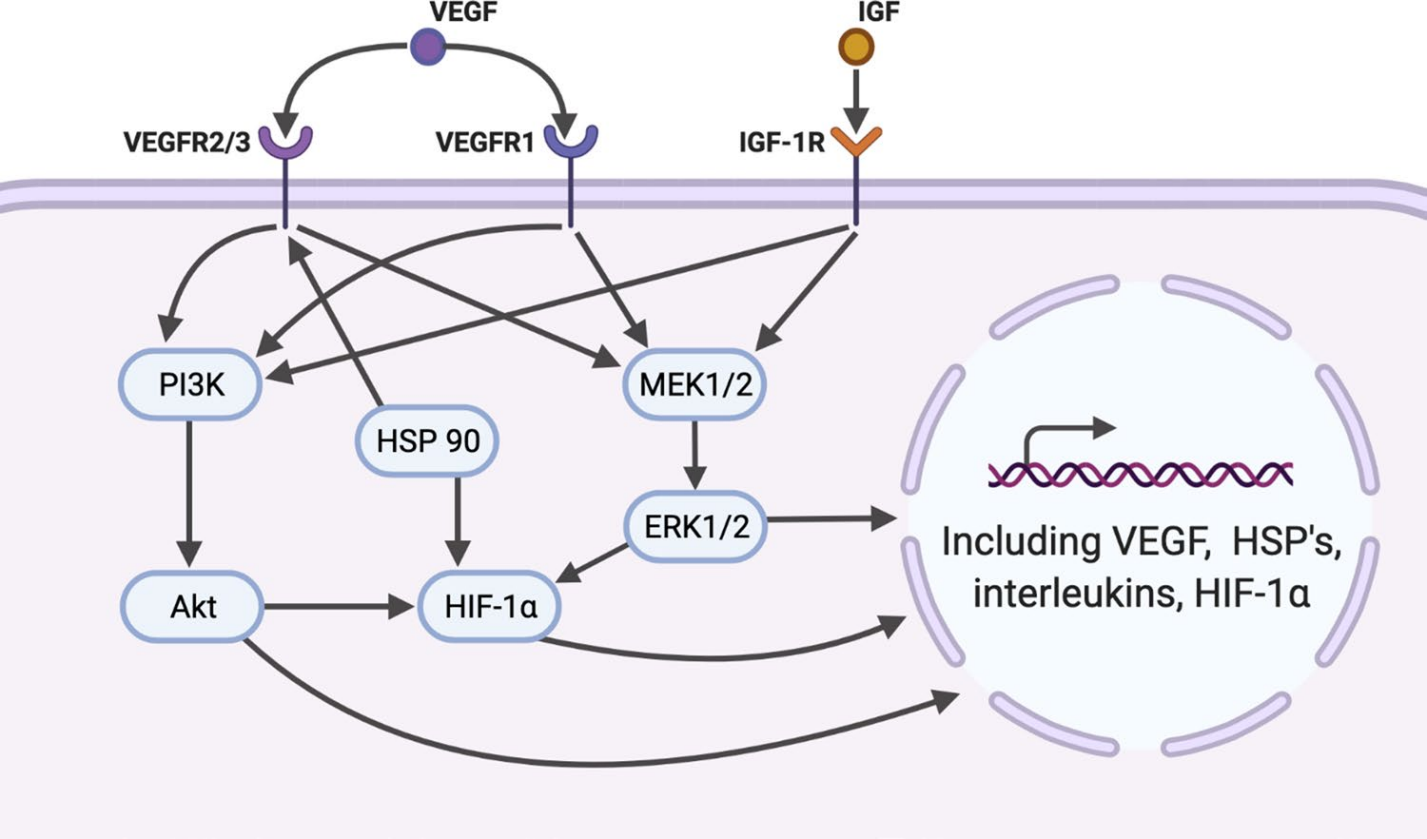
Shared signalling pathways between circulating factors and the proteins they upregulate. Vascular endothelial growth factor (VEGF) and insulin-growth factor (IGF-1) both bind to unique membrane receptors (VEGFR1, VGEFR2/3, and IGF-1R) while sharing common signalling pathways within the endothelium, via the phosphatidylinositol-4,5-bisphosphate 3-kinase (PI3K) and activation of Akt, as well as through the mitogen-activated protein kinase pathway MEK1/2–ERK1/2. These signalling pathways can induce significant upregulation of key proteins including VEGF, heat shock proteins (HSP’s), interleukins, and hypoxia-inducible factor 1 alpha. HSP’s and HIF-1α are also seen to be elevated in response to environmental changes such hypoxia and hyperthermia, and both proteins have to capacity to increase responses to circulating factors via interaction with receptor presentation, signal pathways, or both. Created in BioRender

The importance of intervention-specific study design is made clear when changes in circulating environment are examined in concert with flow. For example, the endothelial cell response to even a small number of inflammatory cytokines (TNFα and IL-1β) is drastically altered when cells are preconditioned under arterial levels of shear stress (Luu et al. 2010). Furthermore, when the pattern of flow is modulated during exposure to TNFα, a significant difference is seen in the response. For example, significant increases in neutrophil adherence are seen when preconditioned cells are exposed to ischemic flow conditions (Sheikh et al. 2003), demonstrating the potential for markedly different responses dependent on blood flow conditions. Alongside this reciprocal relationship between circulating factors and flow, VEGF has the potential to interact with flow responses through its role in the assembly of the p130Cas complex (Evans et al. 2017) and interaction with other mechanosensory signal pathways via binding to VEGF receptors including VEGFR2/3 (Olsson et al. 2006). Adding further complexity still, alongside the interaction between flow and circulating factors, there is also significant potential for interplay between the factors themselves, this is seen in the case of IGF-1 and VEGF as, like many growth factors, they share common signalling pathways through PI3K–Akt and MEK1/2–ERK1/2, resulting in significant overlap in their signalling responses (Fig. 5) (Olsson et al. 2006; Rivera et al. 2011; Bach 2015). Similarly, heat shock proteins, particularly HSP90 (Fig. 5), have been shown to directly interact with HIF-1α and VEGF signalling; through stabilization of HIF-1α (Minet et al. 1999) and increased VEGFR2 receptor presentation (Bruns et al. 2012). Finally, as has been discussed in relation to differences in response to flow (see “Flow-independent adaptation in vivo”), endothelial heterogeneity can also impact endothelial responses to circulating signalling factors, including variability in the magnitude and scope of VEGF-induced MAPK signalling response across BAEC, HUVEC, and HMVEC cell types (Yashima et al. 2001). Taken together, these interactions further emphasise the importance of understanding the complete spectrum of changes seen within the circulation in vivo as well as the collective effects of these changes when modelling endothelial response in vitro.

The development of specific, well-designed cell culture experiments that incorporate core acute intervention factors/responses and better model the physiological environment of these scenarios is key to identifying how changes in circulating environment and flow responses drive beneficial vascular adaptations.

## Current advances and future directions

While we can draw inference as to the vascular effects of non-pharmacological interventions from our understanding of basic and pathological flow responses, more targeted studies are needed to fully understand the mechanisms driving beneficial responses to these interventions. This is particularly pertinent for interventions that share physiological responses with pathological conditions such as passive hyperthermia and ischemic preconditioning. While these interventions mimic the changes seen in disease states, responses are induced over a significantly more transient time-course and in a more controlled fashion, and as a result likely induce different adaptive changes in the longer term (i.e., adaptation vs. maladaptation, as discussed in “Flow-independent adaptation in vivo”).

To improve on our current understanding, interventions should look to mimic the flow profiles seen within a given intervention, utilising the wealth of in vivo data already available on the nature of these responses. As already highlighted (see “Flow-independent adaptation in vivo”), both the magnitude and frequency of pulsatile flow can have an impact on endothelial responses (Zhang and Friedman 2012, 2013) and future research should also consider and look to control the nature of both factors, to better mimic changes in blood flow and heart rate. Similarly, changes in flow should be studied in endothelial cells preconditioned to flow conditions representative of those seen at rest within the vessel of interest in vivo (e.g., 2.0 Pa shear in conduit arteries). These improvements are increasingly possible as a broad array of highly controllable flow culture apparatus are now widely available, both in the form of commercially available equipment and open-source, “do-it-yourself” solutions (Estrada et al. 2011; Byun et al. 2014). An example for the potential of this approach is seen in Wang et al. (2016), in which Matlab-based flow simulations were utilised to demonstrate differences in actin microfilament formation and NO production under resting and exercising flow patterns, based on data taken from in vivo measurements of flow through the common carotid artery.

Physiologically relevant models are also essential in the wider context of environment-based interventions, as both baseline and treatment conditions are often not representative of the conditions seen in vivo, particularly within a healthy population. This is demonstrated in models of hypoxia, with normal/ “normoxic” culture conditions generally set at 21% O_2_ and 5% CO_2_, which provides oxygenation levels well in excess of most physiological settings, and “hypoxic” conditions often at the other extreme, reducing oxygen levels closer to anoxic conditions, rather than those which are physiologically relevant to non-pathological conditions (Al-Ani et al. 2018). Recently, the importance of maintaining and reporting consistent culture conditions in hypoxic experiments has become more prominent within the literature and researchers have begun to focus on developing more physiologically relevant conditions (Pavlacky and Polak 2020) and cost-effective solutions for monitoring changes during experiments (Mathupala et al. 2016). Similarly, the clear differences in endothelium responses dependent on hyperthermic conditions (see Section “Signal cascade responses to changes in culture conditions”) highlights the need for more specific investigation of the responses to changes in environment, particularly in the context of intervention responses compared to pathological conditions, including considering how differences in duration and frequency of exposures may alter adaptive responses. Future studies should also aim to combine the different components of a given stimuli within a single experiment; for example, investigating the endothelial responses to transient elevations in temperature and flow in combination, to understand whether these stimuli offer further benefits over each stimulus in isolation.

The incorporation of samples taken from in vivo experiments when simulating intervention conditions could also improve the physiological relevance of in vitro experiments. Alongside omics-based approaches to better understand the composition of these samples, this approach will also improve our understanding of how changes in the circulating microenvironment may interact with the endothelium, as well as how chronic interventions alter the nature of this microenvironment. This approach was recently taken by Brunt et al. (2018), who demonstrated that ex vivo serum application from participants following 8 weeks of passive heat therapy-induced similar protective benefits in hypoxia-reoxygenation response to those seen in endothelial cells preconditioned at 39 °C. In future, this approach could be utilised not only to model how repeated intervention visits may alter the baseline circulating microenvironment and its impact on endothelial cells in isolation, but also how changes seen within a single visit may alter the endothelial response to concurrent changes in flow, temperature, or oxygen level.

Recent advances in cell culture techniques alongside increased affordability of cutting-edge approaches, such as lab-on-chip systems, are likely to play an essential role in addressing the shortcomings within the current literature, particularly as in vivo research expands beyond large arterial vessels and incorporation of smooth muscle cells. Future research should look to incorporate these advanced culture methods and integrated approaches between in vivo and in vitro research, particularly focussing on the selection of optimal culture conditions and accurate simulation of the magnitude, frequency and duration of intervention responses. This will help to build a more complete picture of the manner in which non-pharmacological interventions interact with the vascular endothelium and ultimately pave the way for the rapid analysis of in vitro endothelial responses in parallel with in vivo research.

## Conclusion

Non-pharmacological interventions have the capacity to drive significant changes in blood flow and, as a result, induce beneficial adaptations within the endothelium. Our understanding of how the cellular mechanosensory mechanisms of the endothelium respond to flow are relatively well developed and, coupled with research into the impact that the circulating microenvironment has on the endothelium, we can begin to interpret how these mechanisms may be altered during interventions. However, research to date has largely focused on resting and pathological scenarios. Targeting future research to address the lack of studies into intervention responses will help to shed light on the potential benefits that non-pharmacological approaches can have on vascular health.
